# Operation CoVER Saint Louis (COVID-19 Vaccine in the Emergency Room): Impact of a Vaccination Program in the Emergency Department

**DOI:** 10.5811/westjem.18325

**Published:** 2024-04-30

**Authors:** Brian T. Wessman, Julianne Yeary, Helen Newland, Randy Jotte

**Affiliations:** * Washington University in Saint Louis, Department of Emergency Medicine, St. Louis, Missouri; † Washington University in Saint Louis, Department of Anesthesiology, Division of Critical Care Medicine, St. Louis, Missouri; ‡ BJC HealthCare, Department of Pharmacy Services, St. Louis, Missouri

## Abstract

**Introduction:**

Coronavirus 2019 (COVID-19) inequitably impacted minority populations and regions with limited access to healthcare resources. The Barnes-Jewish Emergency Department in St. Louis, MO, serves such a population. The COVID-19 vaccine is an available defense to help achieve community immunity. The emergency department (ED) is a potential societal resource to provide access to a vaccination intervention. Our objective in this study was to describe and evaluate a novel ED COVID-19 vaccine program, including its impact on the local surrounding underserved community.

**Methods:**

This was a retrospective, post-protocol implementation review of an ED COVID-19 vaccination program. Over the initial six-month period, we compiled data on all vaccinated patients out of the ED to evaluate demographic data and the impact on underserved regional areas.

**Results:**

We report a successful ED-based COVID-19 vaccine program (with over 1,000 vaccines administered). This program helped raise regional and state vaccination rates. Over 50% of the population that received the COVID-19 vaccine from the ED were from defined socially vulnerable patient populations. No adverse effects were documented.

**Conclusion:**

Operation CoVER (COVID-19 Vaccine in the Emergency Room) Saint Louis was able to successfully vaccinate a socially vulnerable patient population. This free, COVID-19 ED-based vaccine program with dedicated pharmacy support, was novel in emergency medicine practice. Similar ED-based vaccine programs could help with future vaccine distribution.

Population Health Research Capsule What do we already know about this issue?

*The COVID-19 vaccine is an available defense to help achieve community immunity. The ED is a potential societal resource to provide access to a vaccination intervention.*

What was the research question?

*Our goal was to describe and evaluate a novel ED COVID-19 vaccine program, including its impact on the local surrounding underserved community.*

What was the major finding of the study?

*This ED-based COVID-19 vaccine program resulted in over 1,000 vaccines administered.*

How does this improve population health?

*The program helped raise regional/state vaccination rates. Over 50% of those who received the vaccine from the ED were from defined socially vulnerable patient populations.*

## INTRODUCTION

Coronavirus 2019 (COVID-19) first impacted the United States in early 2020. By February 2021, more then 500,000 individuals had died in the US after becoming infected.^1^^–^^4^ Various strategies were employed to limit the spread of the virus including community lockdowns, social distancing, contact tracing, and masking, with varied success and waning adherence over time.^5^ The COVID-19 pandemic inequitably impacted minority populations and regions with limited access to healthcare resources.^6^^–^^8^ The Barnes-Jewish Hospital Emergency Department (BJHED) in Saint Louis, MO, staffed by Washington University School of Medicine in St. Louis emergency physicians with dedicated pharmacy support serves such a population for the bi-state region of Missouri and Illinois.

One of the strongest defenses against this novel virus is vaccines. Development and more widespread distribution of vaccines began in Spring 2021. By September 2021, COVID-19 vaccination was estimated to prevent 56% of expected hospitalizations and 58% of expected deaths.^9^ To achieve community—or “herd”— immunity and thereby reducing the risk of community spread, approximately 67–90% of the population must achieve immunity, either by vaccination or infection. However, vaccine hesitancy, miseducation, and lack of access to vaccines are major barriers to achieving this herd immunity goal.^10^^–^^16^ As the safety net for many communities, the emergency department (ED) provides a multitude of healthcare, educational, and social services.^17^^,^^18^ We hypothesized that the ED could also play a pivotal role with vaccine education, distribution, and access for an underserved population. Prior studies have evaluated the theoretical benefit of using the ED as a potential vaccination resource site.^19^^–^^25^ At the time of project initiation, the state of Missouri ranked nationally in the bottom 10 of states for population vaccination rates, presenting opportunity for improvement.^4^

We started offering COVID-19 vaccines to patients presenting to BJHED on July 21, 2021, initially both the Pfizer and Johnson & Johnson (J&J) vaccines. Of note, this initiation date was well into the delta variant surge of the pandemic. Additionally, vaccination was approved and available for the public through other public health sources. To our knowledge, this free COVID-19 vaccine program by emergency physicians with pharmacy support, based out of an ED, was novel in emergency medicine practice. We named our project: Operation CoVER (COVID-19 Vaccine in the Emergency Room) STL (Saint Louis).

## METHODS

Collaboration on the ED vaccination implementation project between BJHED hospital administration, Washington University emergency physicians, and the pharmacy department began in Spring 2021. On a 24/7 basis, the ED team offers at-the-moment healthcare with confidentiality, patient-centered education, and access to follow-up resources (including completion of the initial vaccination series, if indicated). Such availability differed from other community resources. Barriers throughout the process were identified and resolved. These included Pfizer vaccine storage requirements (ultra-low temperature freezer [−80°C to −60°C]); avoidance of vaccine wastage (as once a vial was diluted, contents had to be used within six hours); hand delivery from inpatient pharmacy to the ED; administration of the vaccine within two hours from vial extraction; record-keeping of appropriate vial lot number; expiration date and injection site; and clinician/nurse training.

Vaccine education was provided to our physicians, nurse practitioners, physician assistants, and nurses. All were encouraged to offer every patient the COVID-19 vaccination. Signage, educational materials, and advertising were developed and distributed to raise awareness of free vaccination access in the ED. Scheduling subsequent doses to complete the initial vaccine series was facilitated by our discharge nurse coordinators. Weekly email reminders tracking vaccines administered were circulated to the Washington University emergency physician/nurse practitioner and physician assistant/resident group.

Based on regional vaccination rates and the healthcare access of our patient population, an assumption was made that approximately one-third of patients would arrive vaccinated. Also considering critical illness/trauma presentation, acute illness, vaccine hesitancy, and clinician forgetfulness, we anticipated another one-third of patients would not be available to consent for vaccination. Of the remaining patients, a vaccination goal rate of 5–10% (approximately 5–15 patients a day) was encouraged. This was discussed at length with pharmacy to support the component of vaccine storage, preparation, and administration in a timely fashion for the ED patient population.

All patients were required to consent to receiving the vaccine, which was documented electronically upon order entry by the clinician. Patients not eligible to receive the vaccine included those with a contraindication to the vaccine, an active COVID-19 infection, or those with a documented COVID-19 infection within the recent past (current recommendation of prior seven-day period). All vaccines were kept in a centralized pharmacy location to meet storage requirements of both the Pfizer and J&J vaccines. Pharmacy staff withdrew doses for the requested vaccine and hand delivered it to the ED bedside nurse along with vaccine vial information (manufacturer, expiration date, lot number, and time of dose withdrawal) and a blank standardized COVID-19 vaccine card (issued by the US Centers for Disease Control and Prevention [CDC]).

The bedside nurse would administer the vaccine dose as soon as possible in view of the two-hour limit between vial withdrawal and administration. Nurses also provided patient education regarding potential side effects and adverse events after receiving the vaccine. Finally, nurses provided each vaccinated patient with education regarding follow-up requirements. Upon BJHED discharge, patients would receive vaccine information sheets and scheduling information for the second vaccine deadline, if applicable. Discharge nurse coordinators would receive a report of all patients who received their first vaccine in the series and would call patients to confirm they had a second vaccine completed or scheduled, as applicable.

This was a retrospective post-protocol implementation review of all BJHED patients receiving the COVID-19 vaccine through Operation CoVER STL between July 1, 2021–January 20, 2022. We report impact on vaccine efforts for various demographics of our region. Data was collected from the electronic health record. We analyzed additional CDC data to compare vaccine regional uptake. Specifically, the Social Vulnerability Index (SVI) was collected from CDC data, specific to our patient population’s affected area. Socially vulnerable populations are especially at risk during a public health emergency due to factors such as socioeconomic status, household composition, minority status, access to transportation, housing type, and lack of resources.^5^^–^^7^ The CDC uses this index to help determine where to leverage healthcare resources to help alleviate human suffering and economic loss (estimate supplies, need for emergency shelters, evacuation planning, required emergency personnel). The SVI database was important during the COVID-19 outbreak to determine which communities would be affected more and require additional support (ie, vaccine implementation).^26^ The data is further divided into quartiles in which quartile “A” represents the lowest/least level of vulnerability and quartile “D” represents the highest/most. We obtained appropriate institutional review board approval (classified as “exempt”) to conduct this retrospective study at our institution.

## RESULTS

**Figure 1.**
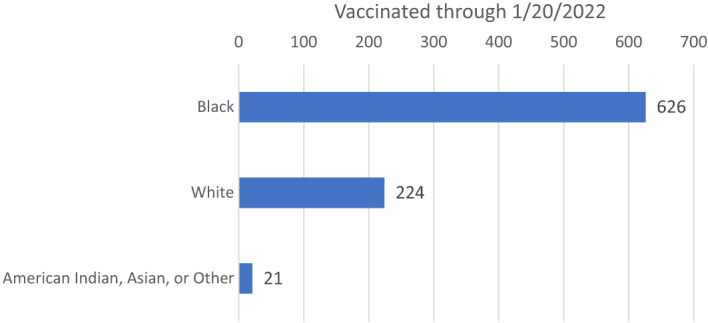
Number of Barnes-Jewish Hospital Emergency Department patients vaccinated by race.

A total of 874 COVID-19 vaccine doses were administered between July 21, 2021–January 20, 2022 (average of 4.78 vaccine doses per day). The total number of impacted patients was 824 individuals. (A minority of patients used the ED for their second vaccine dose administration.) The mean patient age was 44.4 years old (±15.6 years). The distribution in race included 76% (626/824) Black, 27.2% (224/824) White, and 2.91% (24/824) American Indian, Asian, or “other” ethnicity patients (Table 1, Figure 1).

**Table 1.** Demographic data on patients who received the COVID-19 vaccine.

N = 874 vaccines administered; N = 824 patient	
Mean age (years)	44.4 ± 15.6
Mean ED duration (hours)	7.49 ± 5.05
Admitted (yes % [n])	21.6 [189]
Discharged from ED (Yes % [n])	78.4 [685]
Deceased (yes % [n])	0.11 [1]
Mean number of ED visits in prior 5 years	11
Gender (female % [n])	45.3 [396]
Race (% [n])	
Black	76.0 [626/824]
White	27.2 [224/824]
American Indian	0.73 [6/824]
Asian	0.24 [2/824]
Unable to answer	1.94 [16/824]
Mean weight (kg)	82.38 ± 24.1
Mean height (cm)	170.6 ± 10.64
Insurance status	
Self-pay % [n]	29.5 [258]
Insurance % [n]	70.5 [616]
MO Medicaid % [n]	22.3 [195]
MO managed care % [n]	10.1 [88]
Primary care provider	
Yes % [n]	51.4 [449]
No % [n]	48.6 [425]
COVID-19 vaccine given	
Pfizer % [n]	81.1 [709]
Johnson & Johnson % [n]	18.9 [165]
History of +COVID-19 prior to vaccination (Yes % [n])	8.7 [76]
COVID-19+ after vaccination (Yes % [n])	4.9 [43]
Time of vaccine given per shift	
1^st^ shift (0700–1500) % [n]	42.4 [371]
2^nd^ shift (1501–2300) % [n]	30.8 [269]
3^rd^ shift (2301–0659) % [n]	26.0 [227]
Medications given	
EpiPen % [n]	0.23 [2]
Diphenhydramine % [n]	1.03 [9]
Steroids % [n] * (Methylprednisolone, prednisolone, prednisone, or dexamethasone)*	0.80 [7]
Patient address/home states	
Missouri	89.7 [784]
Illinois	9.61 [84]
Indiana	0.11 [1]
Kentucky	0.11 [1]
Mississippi	0.11 [1]
Tennessee	0.11 [1]
Texas	0.11 [1]
Unknown	0.11 [1]
CDC Data	
Missouri data	
Average percentage of the MO population that received 1 dose of any COVID-19 vaccine by 7/21/21	32.9%
Average percentage of the MO population that completed the vaccine series by 7/21/21	28.6%
Average percentage of the MO population that received 1 dose of any COVID-19 vaccine by 1/20/22	45.2%
Average percentage of the MO population that completed the series by 1/20/22	39.0%
County data	
Average percentage of the St. Louis City County population that received 1 dose of any COVID-19 vaccine by 7/21/21	48.2%
Average percentage of the St. Louis City County population that completed the series by 7/21/21	40.8%
Average percentage of the St. Louis City County population that received 1 dose of any COVID-19 vaccine by 1/20/22	69.3%
Average percentage of the St. Louis City County population that completed the series by 1/20/22	55.9%
Average percentage of the St. Louis County population that received 1 dose of any COVID-19 vaccine by 7/21/21	54.9%
Average percentage of the St. Louis County population that completed the series by 7/21/21	48.3%
Average percentage of the St. Louis County population that received 1 dose of any COVID-19 vaccine by 1/20/22	73.1%
Average percentage of the St. Louis County population that completed the series by 1/20/22	61.6%
Social Vulnerability Index (time frame: 7/21/21 – 1/20/22)	
A (0–0.25)	17%
B (0.2501–0.5)	32%
C (0.5001–0.75)	36%
D (0.7501–1.0)	16%

*ED*, emergency department; *MO*, Missouri; *COVID-19*, coronavirus 2019; *CDC*, US Centers for Disease Control and Prevention.

**Figure 2.**
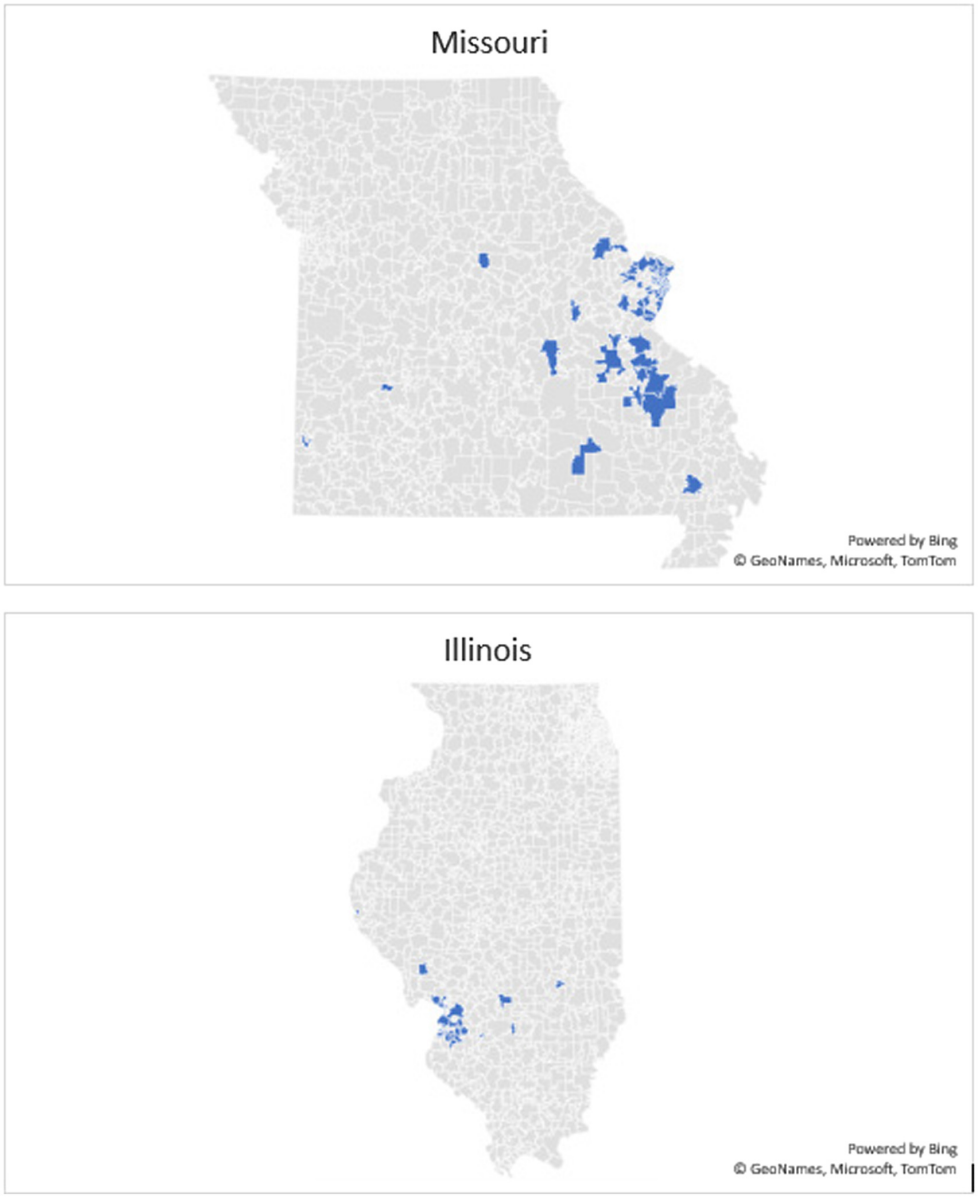
Geographic distribution of vaccinated Barnes-Jewish emergency department patients by listed ZIP code (for states of Missouri and Illinois).

The geographic distribution (based on listed home ZIP code) included 89.7% (784/874) of Missouri patients and 9.61% (84/874) of Illinois patients (Figure 2). Other represented states included Indiana, Kentucky, Mississippi, Tennessee, and Texas. Approximately 22% of vaccinated patients were admitted, and 78% were discharged from the ED. The mean number of ED visits in the prior five years per patient in the vaccinated cohort was 11 total ED visits.

At the time of their BJHED visit and vaccination, 29.5% (258/874) of patients lacked health insurance. Of the 70.5% (616/874) of patients with insurance, 22.3% (195/874) had Missouri Medicaid and 10.1% (88/874) had Missouri Managed Care, both of which provide medical insurance to lower income households. At the time of their BJHED visit, 51.4% (449/874) of patients had a known primary care physician.

**Figure 3.**
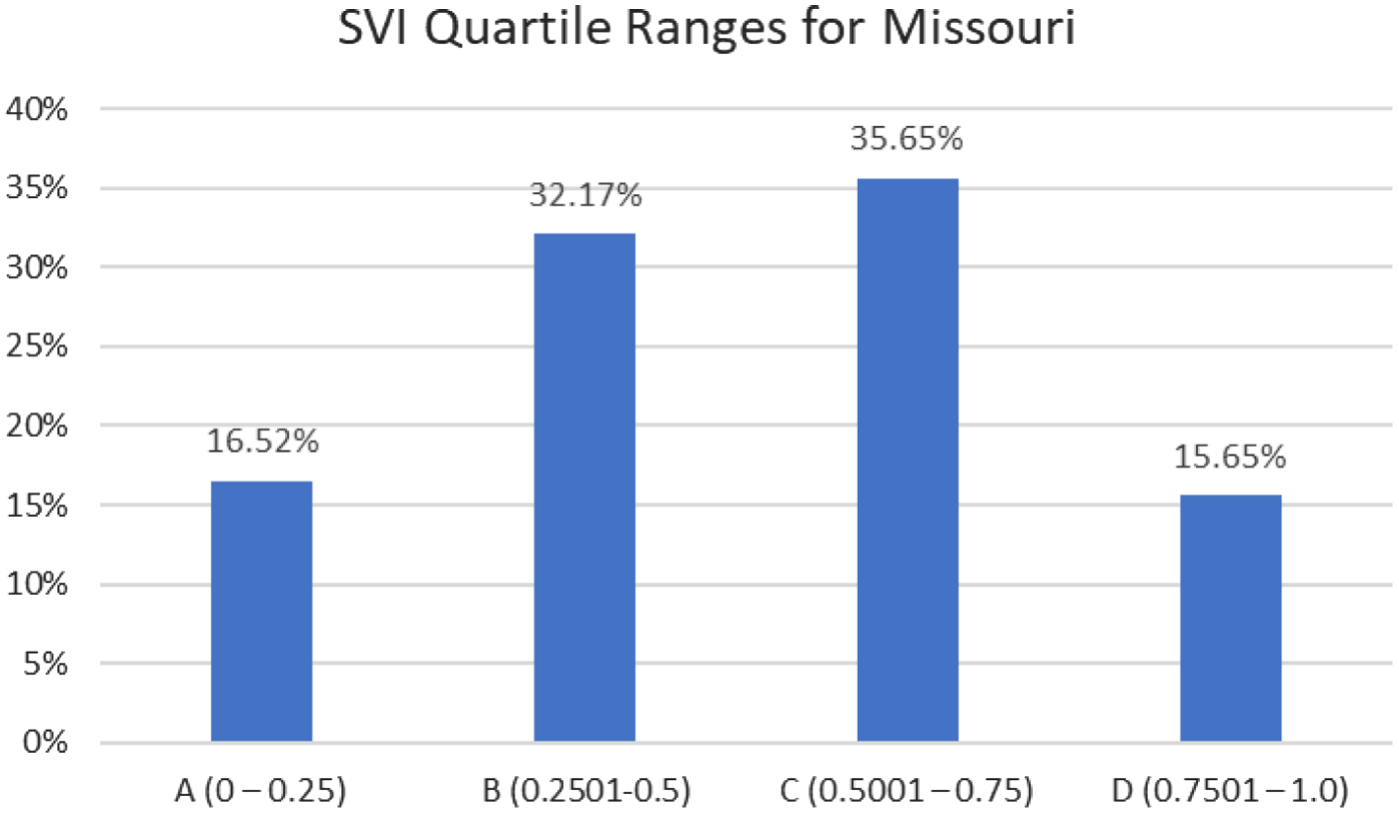
Social vulnerability index for impacted postal codes for vaccinated Barnes-Jewish emergency department patients (A is low/least vulnerable and D is high/most vulnerable).

During the studied time frame, 16% of the patients vaccinated by the BJHED vaccine program lived in areas of high social vulnerability (quartile D of the SVI), with an additional 37% residing in areas of medium-high social vulnerability (quartile C). Altogether, greater than 50% (51.3%) of the patients impacted by the BJHED vaccine administration program were from areas of medium-high and high social vulnerability (Figure 3). See included maps (Figure 2) demonstrating geographic impact on our region (Missouri and Illinois).

Data from Saint Louis City and Saint Louis County (the two largest surrounding regions) showed a 21.1% increase for St. Louis City and an 18.2% increase for St. Louis County for patients receiving at least one dose of the COVID-19 vaccine over the temporal period of Operation CoVER STL. We also reviewed data on adverse outcomes, specifically reviewing all medications provided during each patient encounter. Use of agents for anaphylactic reactions (epinephrine, corticosteroids, antihistamines) were limited in the patient cohort. Two patients received epinephrine 0.3 milligrams intramuscular injections during their ED stay; however, both were unrelated to the vaccine administration (one presented to the ED after an insect sting and another with angioedema as the presenting chief complaint, prior to receiving their COVID-19 vaccine at discharge). We were unable to assess for other potential adverse events such as pericarditis or local site irritation; however, we did not record any repeat visits in this patient cohort for these presenting diagnoses.

## DISCUSSION

Operation CoVER STL is a novel, ED-based vaccination program that meets the needs of an underserved community with a high social vulnerability risk. The Washington University Department of Emergency Medicine serves as the locus of primary care for many of our regional patients. The BJHED census averages 185–240 patients daily, with upward of 80,000 adult patient visits per year. Emergency clinicians are adept at ordering, administering, and documenting vaccines; the most common example is the tetanus, diphtheria, and pertussis vaccine, which is administered almost daily in the ED for open-wound prophylaxis in trauma patients. We have previously been involved with other public vaccination efforts including offering the influenza vaccine in prior “flu seasons,” although with variable success.

The average number of ED visits per patients in this vaccinated cohort was 11 (over the prior five years), demonstrating the unique role the BJHED serves for healthcare in our regional community. Populations within our community are dependent on the BJHED to receive much of their healthcare, reflecting why Operation CoVER STL was impactful. This practice is similar among other large urban areas, with an ED fulfilling the role of central and essential “healthcare” delivery for an underserved patient population.

**Figure 4.**
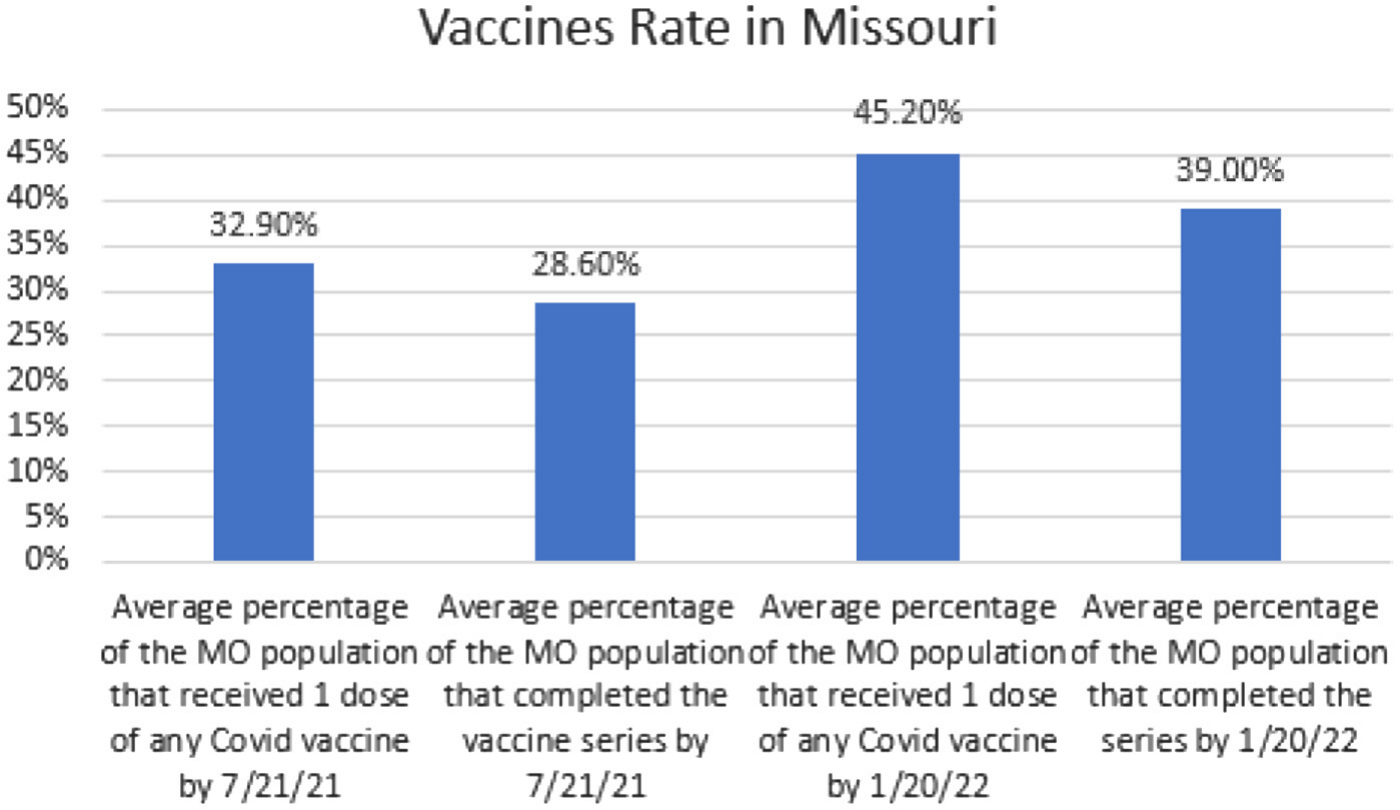
COVID-19 vaccination rates of uptake in Missouri during Operation CoVER STL.

We evaluated CDC data on vaccination rates for COVID-19 vaccine uptake in Missouri during our ED-based initiative. On day 0 of Operation CoVER STL, 32.9% of the state population had received one dose of any COVID-19 vaccine and 28.6% of the state population had completed the COVID-19 vaccine series (two-dose regimen for mRNA vaccines). On January 20, 2022 (end data cohort date), this rate had increased to 45.2% of the state population having received one dose of any COVID-19 vaccine and 39.0% of the population having completed the COVID-19 vaccine series (Figure 4). Programs such as Operation CoVER STL helped along with other initiatives and programs to achieve this 12.3% increase in initial vaccination rates for the Missouri population (16.6% increase in completed vaccination series).

We were able to access data from the CDC to analyze and understand our impact on the region. The SVI data was assessed by the impacted ZIP codes associated to the ED visit. The ZIP codes of our patient cohort were in high-risk socially vulnerable regions, indicating approximately 52% of our vaccination recipients were from socially vulnerable populations. The SVI rates help demonstrate that many patients served by BJHED and Washington University emergency physicians are those with a higher impact from public health emergencies and reside in areas in need of additional support.

We also looked at the racial distribution of Operation CoVER STL efforts. The distribution of vaccines provided in our cohort included 76% Black (626/824 patients) and 27.2% White (224/824 patients). This corresponds to the racial distribution of the geographic area served by the BJHED, which includes St. Louis city and the surrounding bistate regions of Missouri and Illinois. As of 2022, the census of St. Louis city demonstrated that “Black or African American alone” made up 44.8% of the city population and “White alone” made up 46.3% of the city population.^27^ However, our hospital census numbers typically reflect a higher percentage of “Black or African American” patients using the BJHED to access healthcare. During the six-month period of this cohort, the BJHED provided care for 39,570 patients. The racial distribution of the ED population included 61.29% “Black” and 33.92% “White” (Table 2).This may again reflect the role the BJHED serves for specific populations in our city (higher SVI ZIP codes) who are socially vulnerable and why Operation CoVER STL did provide a unique public health resource.

**Table 2:** Racial distribution of Barens-Jewish hospital emergency department population during Operation CoVER STL.

Row Labels	Count of race_primary	Count of race_primary2
American Indian or Alaska Native	0.36%	144
Asian	1.23%	485
Black	61.29%	24254
Declined	0.52%	206
Native Hawaiian or Other Pacific Islander	0.17%	68
Other	0.03%	11
Unable to answer	2.39%	944
Unkonwn	0.09%	34
White	33.92%	13424
(blank)	0.00%	
Grand Total	100.00%	39570

We have also begun to look at clinician attitudes and support of this program through surveys to better understand all parameters of this pilot. We hope our ED-based vaccination program can serve as a model for other EDs with similar socially vulnerable populations.

We have continued to offer Operation CoVER STL through our BJHED. We now offer the Pfizer vaccine and booster(s), if eligible. Of note, we did remove access to the J&J vaccine under CDC public guidance. We have expanded our vaccination efforts to include booster immunizations for eligible patients. As we approached the one-year anniversary of the start of this initiative, we had vaccinated over 1,236 patients as of January 20, 2023).

## LIMITATIONS

This retrospective analysis is not without limitations including its observational nature and our single-center analysis. Vaccinations were given on a clinician-preference basis, and we relied heavily upon clinicians initiating the conversation of vaccines with patients. Of note, the public visual announcements of vaccine access in the ED waiting room, individual patient care areas, and restrooms did lead some patient to initiate the vaccine conversation with Washington Universiy emerg physicians. Vaccine hesitancy was not screened for or assessed in this study, but anecdotally was a common theme limiting vaccine uptake. With the retrospective, blinded design of our data cohort, we were unable to investigate individual factors impacting patient vaccination decisions. On January 20, 2022 (end data cohort date), only 39% of the Missouri population had completed a COVID-19 vaccine series (Figure 4), demonstrating that less than half of our state population had gone forward with a decision to vaccinate. We are aware of multiple emergency clinicians at our institution reporting patients refusing to receive the vaccine when offered as an additional benefit of their ED visit. Our original vaccination goal was set at 5–15 vaccines per day. We ended up administering 4.78 vaccine doses each day; thus, vaccine hesitancy could have impacted our daily rates.

Due to crowding issues, the BJHED has a prolonged wait time and length of stay. It is not uncommon for middle- to low-acuity patients to wait 4-6 hours in the triage area prior to having access to a clinician in an ED room. It is possible that these prolonged ED times could have impacted vaccination rates. Typically, an ED patient arrives with an acute “emergency” chief complaint. Some EDs may have faster evaluation and disposition times, during which time additional requirements (vaccine defrosting, administration) may negatively impact patient flow. However, with a longer ED length of stay, the ED staff may have more opportunities to engage with the patient to discuss specific concerns about vaccine administration. Furthermore, the patient may want to get as many potential available services to maximize care during their prolonged wait. Our large, academic ED has direct access to pharmacy with a dedicated ED clinical pharmacist. Smaller EDs without direct pharmacist access may be limited with a similar vaccine protocol design requiring pharmacy support. Finally, the patient population in our area is primarily urban, potentially limiting applicability to rural areas.

## CONCLUSION

Here we report on the development and implementation of a successful ED-based COVID-19 vaccination program. Our program was able to vaccinate an underserved patient population by meeting the patients where they received their standard healthcare. This program can serve as a model for other emergency departments looking to impact their regions through vaccination efforts. Future studies should evaluate longevity of such programs, as well as public perception and clinician attitudes.
